# Impact of wild prey availability on livestock predation by snow leopards

**DOI:** 10.1098/rsos.170026

**Published:** 2017-06-07

**Authors:** Kulbhushansingh R. Suryawanshi, Stephen M. Redpath, Yash Veer Bhatnagar, Uma Ramakrishnan, Vaibhav Chaturvedi, Sophie C. Smout, Charudutt Mishra

**Affiliations:** 1 Nature Conservation Foundation, 3076/5, IV Cross Gokulam Park, Mysore 570002, India; 2 Snow Leopard Trust, 4649 Sunnyside Av. North, Suite 325, Seattle, WA 98103, USA; 3 Institute of Biological and Environmental Sciences, The University of Aberdeen, 23 St Machar Drive, Aberdeen AB24 2TZ, UK; 4 National Centre for Biological Sciences, TIFR Bangalore 560065, India; 5 SMRU, University of St Andrews, St Andrews, Fife KY16 8LB, UK

**Keywords:** apparent competition, apparent facilitation, conservation conflicts, indirect interactions, predator–prey interactions, snow leopard

## Abstract

An increasing proportion of the world's poor is rearing livestock today, and the global livestock population is growing. Livestock predation by large carnivores and their retaliatory killing is becoming an economic and conservation concern. A common recommendation for carnivore conservation and for reducing predation on livestock is to increase wild prey populations based on the assumption that the carnivores will consume this alternative food. Livestock predation, however, could either reduce or intensify with increases in wild prey depending on prey choice and trends in carnivore abundance. We show that the extent of livestock predation by the endangered snow leopard *Panthera uncia* intensifies with increases in the density of wild ungulate prey, and subsequently stabilizes. We found that snow leopard density, estimated at seven sites, was a positive linear function of the density of wild ungulates—the preferred prey—and showed no discernible relationship with livestock density. We also found that modelled livestock predation increased with livestock density. Our results suggest that snow leopard conservation would benefit from an increase in wild ungulates, but that would intensify the problem of livestock predation for pastoralists. The potential benefits of increased wild prey abundance in reducing livestock predation can be overwhelmed by a resultant increase in snow leopard populations. Snow leopard conservation efforts aimed at facilitating increases in wild prey must be accompanied by greater assistance for better livestock protection and offsetting the economic damage caused by carnivores.

## 1. Introduction

The global food economy has increasingly shifted towards livestock products, and a growing proportion (1.4% per year; total 752 million in 2012) of the world's poor (2.6 billion people living on less than 2 US$ a day) rear livestock today [1]. Over one third of the global land area is used to graze livestock [2]. Pastoralists, who graze their livestock in rangelands, produce an estimated 10% of the world's meat, and support over 200 million pastoral households [3]. Predation on livestock by wild large carnivores is a threat to agricultural security, with up to 3% of local livestock holdings lost to carnivores in sites in North America and Europe, and up to 18% in Africa and Asia [4]. Individual families may lose up to 50% of their average *per capita* income to livestock predation by carnivores, which can sometimes be high enough to keep affected people below national poverty lines [5–10]. Further, the livelihoods in pastoral economies are always under threat of environmental or climatic hazards such as droughts, flood and extreme winters which have additive or compensatory impact alongside predation by carnivores [11].

**Figure 1.**
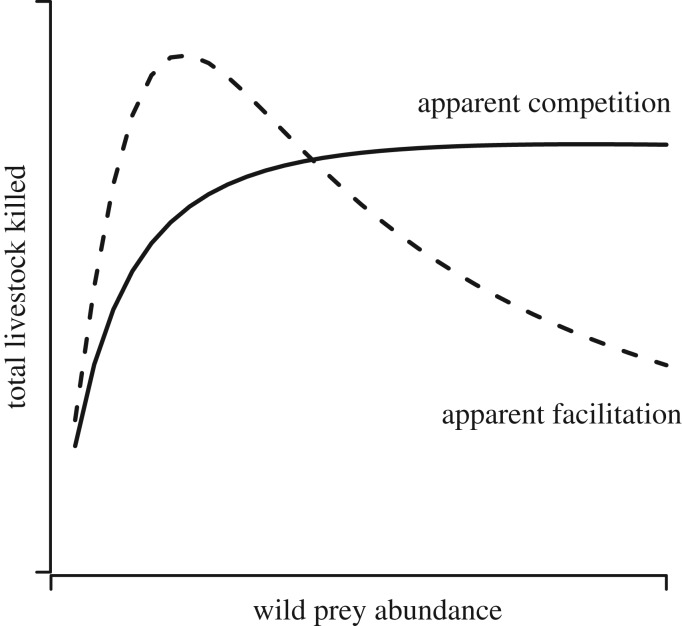
Schematic representation of apparent competition and apparent facilitation between livestock and wild prey, mediated by a predator. We tested these two hypotheses using snow leopards *Panthera uncia* and their mountain ungulate prey as our model system. The solid line indicates the trend in livestock predation when the numerical response to wild prey is type I and functional response is type II (non-switching). The resultant pattern is called apparent competition when the increase in the population of one prey species (wild prey) increases predation of the second prey species (livestock). The dotted line indicates the trend in livestock predation when the numerical response is type I and functional response is type III (switching). The subsequent decline in livestock predation with an increase in wild prey is termed ‘apparent facilitation’. The initial increase in livestock predation is predicted by both hypotheses because carnivore populations are assumed to be zero at zero wild prey abundance.

Conservation programmes that include ways of sharing and offsetting economic losses to farmers are likely to help the communities bearing the disproportionate costs of carnivore predation [12], although they sometimes lead to over-reporting of predation events [13]. A considerable proportion (63%) of large terrestrial carnivores (more than 20 kg, order Carnivora) are threatened with extinction today [14]. Persecution by farmers over perceived livestock depredation behaviour has already caused the extinction of two large-carnivore species; the Falkland wolf *Dusicyon australis* and marsupial wolf *Thylacinus cynocephalus* [15] and remains an important threat to at least 85% of the extant species of large terrestrial carnivores [14]. Efforts to increase the abundance of wild prey populations are often recommended as a policy measure to reduce carnivore predation on livestock [16–21]. However, the impact of wild prey availability on predation patterns of large carnivores is debatable [22–24]. As the density of wild prey increases, predators may consume more of them, resulting in reduced predation on livestock; a mechanism termed ‘apparent facilitation’ (figure 1) [25,26]. Alternately, greater wild prey availability can lead to increases in the density of the predator, which in turn can result in increased predation on livestock; a mechanism called ‘apparent competition’ (figure 1) [27,28]. Theory therefore predicts that, ultimately, the outcome of such a policy measure to increase wild prey abundance will depend on the shape and the strength of the influence of prey density on predator density (numerical response) and predator diet (functional response) [29]. Numerical response is defined as the change in predator density as a function of prey density while functional response is the change in predator's intake rate as a function of prey density [30,31]. In the context of the conservation and management of large mammal populations, such indirect interactions have received little empirical investigation [32], let alone their policy implications.

Wild prey density is known to be a critical determinant of large-carnivore density [33,34]. There is little information available, however, on the functional responses of large carnivores in the wild, with the exception of the grey wolf *Canis lupus* which is known to show both type II and type III responses to wild ungulate prey [35]. Type II and III functional responses respectively represent asymptotic and sigmoid-shaped relationships between the rate of prey consumption and prey density.

In this paper we ask whether an increase in the density of wild ungulate prey would reduce or increase the number of livestock killed by large carnivores. Our focus is the endangered snow leopard *Panthera uncia*. The species occurs across the mountain ranges of Central Asia. Its survival is threatened, among other causes, due to persecution over its livestock-killing behaviour [16,36]. Livestock contribution to snow leopard diet is variable [37], and has been reported to be as high as 70% [38,39]. Facilitating the recovery of wild ungulate prey of the snow leopard is considered an important measure to reduce the extent of livestock depredation [16]. However, the potential impact of this management strategy on livestock predation rates is not understood.

Theory predicts that if snow leopards prefer wild ungulates but their population does not increase in response to increased wild prey (numerical response), we expect livestock predation by snow leopards to decrease as wild prey increases (apparent facilitation). On the other hand, if snow leopard density increases as a function of wild prey density, then, depending on the strength of the functional response, livestock predation by snow leopards could increase and stabilize (apparent competition) or increase and then decline (apparent competition followed by apparent facilitation) with type II and type III functional responses, respectively.

We assessed the numerical and functional responses by examining the density and diet of snow leopards at seven sites in Asia representing a gradient of wild prey to livestock density. Based on the results, we modelled the impact of increasing livestock and wild prey density on the extent of livestock predation. Our work has policy implications for conservation and livestock management in Central Asia and the extensive rangeland systems in other parts of the world where farmers and large carnivores coexist.

## 2. Material and methods

### 2.1. Study sites

We sampled at seven sites in the Trans-Himalayas and Altai. These sites were Kibber (32.36° N; 78.01° E), Lingti (32.32° N; 78.33° E), Lossar (32.41° N; 77.63° E), Pin (31.89° N; 77.94° E) and Tabo (32.06° N; 78.32° E) along the Spiti Valley in Himachal Pradesh, India; Rumtse (33.72° N; 77.76° E) in Jammu and Kashmir, India; and Tost (43.17° N; 100.46° E) in South Gobi, Mongolia. The areas and effort are summarized in table 1. Maps of the study sites have been included in the electronic supplementary material, appendix A. The wild ungulate prey species included blue sheep *Pseudois nayaur* in four sites, ibex *Capra sibirica* in two sites, ibex and argali *Ovis ammon* in one site, and blue sheep and urial *O. vignei* in one site. The primary domestic ungulates were horse *Equus caballus*, cattle *Bos taurus*, sheep *O. aries* and goat *C. hircus* in seven sites, yak *B. grunniens* and donkey *E. asinus* in six sites, and Bactrian camel *Camelus bactrianus* in one.

**Table 1.** Total effort for collecting scat samples at the seven study sites and amplification success with species-specific primer and microsatellite loci.

site	area (km^2^)	distance walked (km)	no. transects	scats collected	snow leopard (species-specific primer)	amplification for all seven loci
Lingti	240	122	24	53	41	15
Lossar	219	106	21	50	10	3
Kibber	411	133	28	44	30	15
Pin	270	110	22	24	16	2
Tabo	341	131	26	46	31	10
Rumtse	300	117	23	43	28	8
Tost	250	132	27	45	35	n.a.^a^

^a^Snow leopard population at Tost was estimated using camera trapping.

### 2.2. Estimating wild prey density

Wild prey population was estimated using the double observer survey method [40]. Details of the survey at the five sites of Kibber, Pin, Lingti, Tabo and Lossar have been described in [40]. The study area was divided into three and eight survey blocks for Rumtse and Tost, respectively. Rumtse and Tost were surveyed over 3 and 8 consecutive days, respectively. Rumtse was surveyed in November 2010 and Tost was surveyed in November 2011. The other five sites were surveyed between March and June 2010.

### 2.3. Estimating livestock density

Livestock population was censused in all the villages and herder camps in Kibber, Lingti, Lossar, Pin, Tabo and Tost sites. We interviewed all the people responsible for herding the livestock in all the villages within our study sites to record the age-wise abundance of all livestock species. For the Rumtse site, data on population of livestock were collected from the headman who maintains a record of livestock holding in all the four villages using the area. All livestock census were conducted during or immediately after the wild prey surveys.

### 2.4. Estimating snow leopard diet and density

#### 2.4.1. Snow leopard scat collection protocol

Scats were collected from trails laid along prominent snow leopard habitats such as ridge-lines and cliff bases. The trails were distributed across the study area to achieve a uniform spatial coverage and to avoid any large ‘holes’ where a snow leopard had a zero probability of detection (table 1) [41]. Scat sampling in Kibber, Lingti, Lossar, Pin, Rumptse and Tabo was completed between August and November 2010. Sampling in Tost was conducted in November and December 2011. On encountering a scat, we recorded information on the size of the scat (length and diameter), GPS location, presence of snow leopard pugmark, scrapemarks or spray, the strata on which the scat has been deposited, the general microhabitat and any other general remarks. Only scats that were likely to belong to snow leopards were collected. We avoided collecting old and disintegrating scats. The scat samples were carefully collected to avoid contamination and stored in absolute alcohol. DNA was extracted following [41] and the details of DNA extraction, microsatellite primer selection and PCR and genotyping are explained in electronic supplementary material, appendix A.

#### 2.4.2. Species identification from scat

A total of 305 potential snow leopard scats were collected across the seven sites out of which 191 (62.62%) amplified with our snow leopard specific primer. The rest were possibly too old and degraded or belonged to other sympatric carnivores. Site-wise details are included in table 1.

#### 2.4.3. Identification of individual snow leopard from scat

We were able to genotype 53 (34%) snow leopard positive samples on all seven loci. The number of alleles per locus varied from 2 to 4. Our analysis with the program CERVUS resulted in the probability of identity or PID values which was calculated to be 0.000089 (Unbiased) and 0.012 (Siblings). Site-wise details are included in electronic supplementary material, appendix A, table S1.

#### 2.4.4. Individual identification and abundance

We then compared these genotype profiles using the identity analysis module in program CERVUS [42], which identifies samples with identical genotypes for the specified number of loci. Identical genotype profiles at all the seven loci were used to identify multiple instances of the same individual. This analysis allowed us to discern both the number of unique individuals and the number of recaptures. We used *Capwire* [43] to estimate the abundance of snow leopard at each site. We used the even capture probability model (ECM). The ECM assumes equal capture probability for each individual. We expect this to be true in our case as all transects were equally spaced from each other. Snow leopard density was estimated by dividing the abundance estimate by the size of the study site.

#### 2.4.5. Camera trapping (one site; Tost)

The study area was divided into ten equal grids of approximately 25 km^2^. One Reconyx RM45 or HC500 camera trap was deployed per grid at the most suitable location. This was part of a larger camera-trapping study which covered the 1684 km^2^ around our study area [44]. We could identify the individual snow leopard in 96% of the independent photo captures. Individuals were identified using unique markings [45]. For consistency, data were analysed using *Capwire* [43] such that each camera trap capture was considered as an independent sample.

Each of the seven study sites was sampled once. The livestock census, double observer surveys for wild ungulate abundance and snow leopard scat sampling within each site were done within the same month.

### 2.5. Snow leopard diet

Scat samples confirmed to be from snow leopards using the molecular technique were analysed for diet using the micro-histological method [18,46]. We collected reference hair samples of all the potential prey of the snow leopard. A minimum of three reference slides were prepared per animal per site. Hair remains from snow leopard scat samples were used for prey species identification. Shape, size, colour and structure of the cuticle and medulla were used in identification. We examined 10 hairs from each scat sample at random. We recorded the relative occurrence of each prey species in snow leopard diet, i.e. the proportion of hair samples of a particular prey species to the entire hair sample positively identified. Asymmetric 95% confidence intervals were calculated through Monte Carlo simulations with a scat as a sampling unit with 1000 permutations using random draws from the observed distribution with replacement. We estimated the biomass of each species consumed using the Ackerman's linear biomass model [47]. The functional response modelling was conducted with both frequency of occurrence and biomass. The results were identical and hence we only present the frequency of occurrence, which is a more parsimonious measure.

### 2.6. Numerical response analysis

To assess the shape of snow leopard numerical response, we estimated its density at seven sites along a gradient of density of its wild ungulate prey (ibex *Capra ibex*, argali *Ovis ammon*, urial *Ovis vignei*, and blue sheep *Pseudois nayaur*). We fitted linear equations with snow leopard density as a function of wild prey and livestock density separately. We used software R v. 2.14.1 to fit the models.

### 2.7. Functional response analysis

To assess if snow leopards preferred wild prey over livestock and to examine the shape of its functional response, we used a general multi-species functional response model [48] that describes diet composition and relative prey preference:

2.1 $$f_{i}=\frac{{\left(a_{i}n_{i}\right)}^{m}}{{1+h\Sigma_{j}(a_{j}n_{j})}^{m}},$$

where, *f*_*i*_ is the consumption rate of prey type *i*, *n*_*i*_ is the abundance of prey type *i*, *a*_*i*_ and *h* are constants referring to the preference for prey type *i*, and the handling time, respectively, *j* is the total number of species, while *m* is the switching parameter. Values of *m* significantly larger than 1 indicate prey switching and larger values of *m* suggest stronger switching [49,50].

From equation (2.1) we derived an equation that describes relative diet composition. The ratio of prey consumed:

2.2 $$\frac{f_{L}}{f_{W}}={\left(\frac{cn_{L}}{n_{W}}\right)}^{m},$$

where, subscripts L and W denote livestock and wild prey, respectively. The ratio of *a*_L_ and *a*_W_ is represented by *c*, which is a measure of the bias in the predator's diet towards one prey species, or, in other words, indicating preference towards that prey species [51]. Values of *m* and *c* were estimated by fitting equation (2.2) to the data on snow leopard diet and abundance of livestock and wild prey using Bayesian methods with uninformative priors [29]. Fitting was carried out using WinBUGS [42] and the code is provided in electronic supplementary material, appendix B. We assumed that the snow leopards in the study obtained sufficient food (livestock and wild prey) to satisfy their energetic requirements. We use symbol *Q* to represent this baseline consumption rate.

2.3 $$f_{L}+f_{W}=Q.$$

Equations (2.2) and (2.3) give the consumption rate due to a single leopard as:

2.4 $$f_{L}=\frac{Q{\left(cn_{L}\right)}^{m}}{{\left(cn_{L}\right)}^{m}+{\left(n_{W}\right)}^{m}}.$$

For this exercise, we assumed an annual kill rate (*Q*) of 45 ungulates (domestic and wild) per snow leopard. Previous studies [18,52] have considered *Q* to lie between 20 and 30 ungulates based on assumptions regarding the daily food requirement of a snow leopard-sized carnivore, while more recent data based on monitoring actual kills made by radio-collared snow leopards has yielded an estimate of *ca* 45 kills per year [53].

### 2.8. Modelling impact of wild prey population on livestock depredation by the predator

To find the rate of prey consumption by the whole population of leopards (denoted as *k*) we multiply expression (equation (2.4)) by the leopard population *n*_SL_ calculated from the fitted numerical response.

2.5 $$n_{\mathrm{SL}}f_{L}=k=\frac{Q{\left(cn_{L}\right)}^{m}(an_{W}+b)}{{\left(cn_{L}\right)}^{m}+{\left(n_{W}\right)}^{m}},$$

where *a* and *b* are constants and *n*_SL_ represents the population of snow leopards in the area. We assumed that snow leopards were able to satisfy their energetic requirements and consumed 45 large ungulate prey per year. Based on the predictions of our diet model for the proportion of the yearly diet represented by each prey type we were then able to estimate *k.*

## 3. Results

### 3.1. Wild ungulate and livestock population estimation

**Figure 2.**
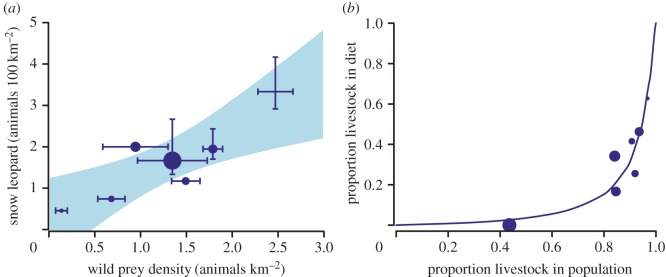
Relationship between snow leopard density and wild prey density (*a*). Error bars in (*a*) indicate 95% CI of the estimates. Shaded area in (*a*) indicates the 95% CI of the significant regression model (slope = 1.01 (s.e. = 0.27); intercept = 0.23 (s.e. = 0.39); *R*^2^ = 0.76; *p* = 0.01). Relationship between the proportion of livestock in snow leopard diet and relative density of livestock vis-à-vis wild ungulates. The size of the dot indicates the livestock population at the site (range 1.9–10.8 livestock per km^2^) (*b*). The curve depicts the fitted model of snow leopard functional response based on equation (2.2). The size of the dot indicates the density of wild prey at the site (range 0.14–2.47 per km^2^).

Wild ungulate densities ranged from 0.1 km^−2^ at Lossar to 3.1 km^−2^ at Lingti (figure 2*a*). The detection probability for the double observer surveys ranged from 0.6 to 0.8. Livestock density ranged from 1.9 km^−2^ in Lingti to 19.5 km^−2^ at Rumptse (electronic supplementary material, appendix A).

### 3.2. Numerical response

Snow leopard densities ranged from 0.46 to 3.30 individuals 100 km^−2^ across our seven study sites, and increased as a linear function of wild prey density (*R*^2^ = 0.76 *p* < 0.01; figure 2*a*); by 1.01 individuals 100 km^−2^ for a unit increase in wild prey density km^−2^ (figure 2*a*; table 2). In contrast, snow leopard density did not show any discernible relationship with livestock density (slope = 0.01; *p* = 0.85).

**Table 2.** Estimated parameter values for the numerical and functional responses. Numerical response was estimated by fitting a linear regression model to snow leopard and prey density estimates. Functional response parameters were estimated by fitting generalized multi-species functional response model to the data on snow leopard diet and abundance of livestock and wild prey using Bayesian methods with uninformative priors [51]. These estimates were then used for further simulations.

	numerical response		functional response	
	slope (b)	intercept (a)	bias in prey selection (c)	switching parameter (m)
parameter estimate	1.01	0.23	0.056	1.15
standard error	0.27	0.39	0.004	0.07

### 3.3. Functional response

Wild prey contribution to snow leopard diet ranged from 35% in Lossar to 95% in Lingti. Contribution of small mammals was less than 4% across all the sites. Snow leopards preferred wild prey as shown by a relatively low value of *c* (0.056, CI 0.049–0.064) [20]. The diet results have been summarized in table 3. The model fit was good with a high *r*-squared (RMSE = 0.08; *R*^2^ = 0.82). Snow leopards showed a type III functional response [49,50], with the mean estimated *m* being significantly higher than 1 (at 1.148, CI 1.013–1.326; figure 2*b*), although the strength of the sigmoidal response was weak [49,50]. The parameter estimates for numerical and functional response have been summarized in table 2.

**Table 3.** Per cent contribution of wild ungulates and livestock species to snow leopard diet at seven sites. n.a. indicates that the species was not available.

site	blue sheep	ibex	argali	yak	horse	camel	goat/sheep	cattle	donkey	small mammals	unidentified
Kibber	48	18	n.a.	10	10.6	n.a.	10.2	0	0	0	3.2
Lingti	95	n.a.	n.a.	0	0	n.a.	0	0	0	2	3
Lossar	n.a.	34.6	n.a.	0	23	n.a.	0	10	26.9	2.3	3.4
Pin	n.a.	55.9	n.a.	0	14.2	n.a.	12.4	6.2	6.8	0	4.3
Rumtse	51.5	n.a.	n.a.	0	3.5	n.a.	40	0	0	3.5	1.5
Tabo	83	n.a.	n.a.	0	6.5	n.a.	6.5	3.3	0	0	1
Tost	n.a.	65	4.4	n.a.	1.8	3.7	20.6	0	n.a.	2.9	3.8

#### 3.3.1. Modelling impact of wild prey population on livestock predation

**Figure 3.**
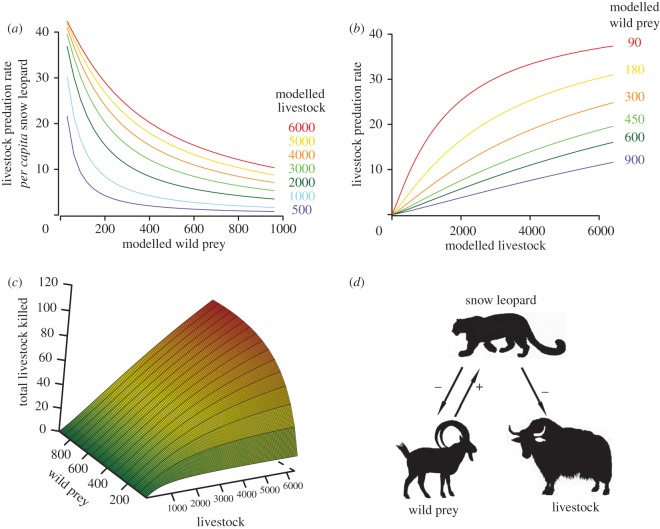
Predicted annual rate of livestock predation by an individual snow leopard along a gradient of wild prey density based on the simulation model (*a*). Predicted annual rate of livestock predation by an individual snow leopard along a gradient of livestock density based on the simulation model (*b*). Total number of livestock predicted to be killed by snow leopards (per year) along gradients of livestock and wild ungulates based on the simulation model (*c*). Schematic relationship of our findings on snow leopard diet, their density, and the density of livestock and wild ungulates (*d*). The positive sign indicates the numerical relationship between wild prey and snow leopards. The negative sign indicates the impact of snow leopard predation on the population of livestock and wild ungulates. The strength of the impact of predation depends upon the relative density of livestock and wild ungulates.

We found that the rate of livestock killing ( *f*_L_) by an individual snow leopard increased with increasing livestock, but declined with wild prey (figure 3*a*,*b*). However, the total number of livestock killed per year, modelled as a product of the total number of snow leopards and *f*_L_ (equation (2.4)), increased with increase in both wild prey and livestock (figure 3*c*). The total livestock killed per year initially with wild prey, ultimately reaching saturation (figure 3*c*).

## 4. Discussion

Snow leopard density in our study sites increased as a linear function of the wild prey population (type I numerical response). Our data suggest that wild prey are a primary determinant of snow leopard density and critical for conservation of this endangered species. Although we expect snow leopard density to ultimately saturate with wild prey density as other factors such as denning sites or territoriality become limiting, we did not find any evidence for saturation. This finding is consistent with studies on other carnivores [33,34]. The numerical response of snow leopards is consistent with their prey selection, as there was also strong selection for wild prey, as indicated by the bias parameter *c* in the functional response modelling. Our results show that despite livestock contributing as much as 62% to snow leopard diet (site Lossar), wild prey remains the preferred prey and the critical determinant of the snow leopard density. As shown by the lack of relationship between livestock and snow leopard abundances, livestock by itself is unable to sustain snow leopards.

Our functional response modelling of snow leopard diet showed that the switching parameter *m* was significantly greater than 1, though not very much higher in magnitude (*m* = 1.14). Thus, snow leopards showed a type III functional response, but the strength of the response was weak; i.e. snow leopards seem to have the ability to switch, but they continue to take both prey even at high density of wild herbivores (electronic supplementary material, figure S1 in appendix A summarizes other possible outcomes for different estimates of *m* and *c*).

Together, the numerical (type I) and functional responses (weak type III) suggest that livestock predation by snow leopards would increase and then stabilize (apparent competition) with an increase in wild prey (figure 3*c*). Snow leopard diet across a gradient of wild prey to livestock ratios showed that livestock contribution increased sharply when livestock population outnumbered wild prey populations by a ratio of four to one.

Our results support the idea that facilitating an increase in wild ungulate populations is a critical measure for increasing carnivore populations [33,34]. This is an increasing challenge, as Himalayan and Central Asian mountain landscapes are becoming increasingly livestock dominated. In fact, wild herbivore biomass has been reduced to less than 5% of the livestock biomass in much of Central Asia [54]. This problem is made more challenging by our finding that increases in wild ungulates also mean increases in livestock predation, especially when livestock are abundant. Although the rate of livestock predation per snow leopard declined with an increase in wild prey, the total number of livestock killed increased due to the increased snow leopard density. Our model predicts highest livestock predation by snow leopards in areas with high density of livestock as well as wild prey. While livestock at high density can out-compete wild prey in these regions [54], it appears that these two groups can also coexist at relatively high density in areas of higher plant productivity. One such site was Kibber which had the second highest density of livestock as well as wild prey (electronic supplementary material, appendix A, table S2).

We recognize that our seven study sites represented a relatively small sample size from a statistical point of view. However, datasets of this size are rare even for relatively well-studied wide-ranging large carnivores such as tigers (*Panthera tigris*) [34], and have so far been unavailable for the endangered snow leopard that occurs at low densities in harsh mountainous terrain. While inter-site differences cannot be ruled out, we consider our sites to be representative of snow leopard habitats across the mountains of Asia. There was sedentary pastoralism in six sites, nomadic pastoralism in four sites and three sites had both. There were at least four species of livestock available at each site (table 3), ranging from large-bodied and free-ranging animals like yak, camel and horses to small-bodied herded animals like sheep, goat and cow (electronic supplementary material, appendix A table S2). Three sites had at least two species of wild prey (Tost, Rumtse and Kibber) while the other four sites had only one species of wild herbivore (table 3). This diversity of livestock and wild prey species together with a diversity of herding practices allow us to generalize to other areas.

The size of our study sites was equal to the home range of one to three snow leopards (Snow leopard home range estimated at 150–900 km^2^) [55]. Thus the increase in snow leopard density that we have demonstrated could have been a result of foraging patch selection (an aggregation response) rather than a true numerical response. However, this does not affect our primary finding that snow leopard density scales linearly with wild prey density and the conclusion that wild prey is the critical determinant of snow leopard population. Also, the primary prediction of our model that livestock predation by snow leopards will increase and then stabilize with an increase in wild prey density remains unchanged irrespective of the mechanism of increase in snow leopard density (numerical or aggregation response). A recent study showed that the relative habitat use of the snow leopard increased linearly with the realized values of wild prey density [56].

Our findings are particularly apposite given the predicted increase in global livestock production, which is especially rapid in the developing world [57,58]. The global food economy has increasingly shifted towards livestock products, and a growing proportion of the world's poor rear livestock today [1]. Conservation will have to recognize this trade-off that increasing population of wild prey will also increase the impact of carnivores on livestock production. For livelihood security of local people, carnivore conservation efforts, while focusing on enhancing wild ungulate populations, must be accompanied by greater assistance for better livestock protection and offsetting carnivore-caused economic damage.

## Supplementary Material

Appendix A: DNA extraction and PCR protocol

## Supplementary Material

Appendix B: Code for the Bayesian analysis of the functional response

## Supplementary Material

Appendix C1: Data for functional response analysis

## Supplementary Material

Appendix C2: Data for functional response analysis

## Data accessibility

Necessary data and code are provided in electronic supplementary material, appendices B and C. All the primary data are available at the Dryad Digital Repository. doi:10.5061/dryad.8p689 [59].
