# Supplementary material for: Impact of wild prey availability on livestock predation by snow leopards
Source: R Soc Open Sci. 2017 Jun 7;4(6):170026. doi: 10.1098/rsos.170026 (PMC5493907; doi:10.1098/rsos.170026)
Supplement: Appendix A: DNA extraction and PCR protocol [file rsos170026supp1.doc]

**Appendix A**

*DNA extraction and species identification*: DNA was extracted from the scat samples in a separate, UV irradiated pre-PCR laboratory space using the commercially available QIAamp DNA Stool mini kit (QIAGEN Inc.) following the manufacturer’s instructions with slight modifications (Mondol et al. 2009). All extractions (in sets of eleven samples) included a negative control to monitor contamination. The DNA samples were then amplified using the snow leopard specific primer (UNC-1). This primer amplifies 111 base pairs of the NADH2 gene and was found to be specific to the snow leopard when tried with other sympatric carnivores and prey species. This ensured that only genuine snow leopard samples were used for further analysis.

*Microsatellite primer selection*: We adopted seven microsatellite loci characterised for snow leopards from (Janeka et al. 2008*,* Mondol et al. 2012) based on their polymorphic information content, amplification success with field collected fecal samples, and low error rate.

*PCR and Genotyping*: Amplification was carried out in 10 μl reaction volumes containing 4 μl Qiagen multiplex PCR buffer mix (QIAGEN Inc.), 0.2 μm labelled forward primer (Applied Biosystems, Carlsbad, CA, USA), 0.2 μm reverse primer, 4 μm BSA and 3 μl of the template DNA and 1μl of ultrapure distilled water. The temperature regime included an initial denaturation (94°C for 15 min); 45 cycles of denaturation (94°C for 45 secs), annealing (Ta for 45 secs) and extension (72°C for 45 sec); followed by a final extension (72°C for 30 min) in an Eppendorf thermocycler. We incorporated PCR negatives in all reactions to check any cross contamination. One microlitre of the amplified product was added into 12 μl of formamide (Applied Biosystems) and 0.5 μl GeneScan –500 LIZ ® size standard (Applied Biosystems) then run into an automated sequencer ABI3100XL (Applied Biosystems). Microsatellite alleles were scored with GENEMAPPER version 4.0 (Applied Biosystems). To get reliable genotypes, we followed the multiple tube approach by (Taberlet 1996) and repeated the PCR, genotyping and scoring process four times. Samples with consistent results for at least three repeats were considered for further analysis. This implies that quality index of 0.75 was used following (Miquel et al. 2006).

Table S1: Locus specific information on genetic variability and error rates

| **LOCUS** | **Allele size** | **Dropout** | **False Alleles** | **HObs** | **HExp** | **No of**  **Alleles** |
| --- | --- | --- | --- | --- | --- | --- |
| PUN 82 | 110-115 | 5.32 | 2.08 | 0.666 | 0.509 | 2 |
| PUN 100 | 89-97 | 1.39 | 0.48 | 0.189 | 0.47 | 4 |
| PUN 124 | 93-103 | 1.23 | 0.82 | 0.208 | 0.411 | 3 |
| PUN 132 | 113-123 | 0.63 | 2.25 | 0.642 | 0.543 | 3 |
| PUN 229 | 106-110 | 1.69 | 1.74 | 0.717 | 0.628 | 3 |
| PUN 337 | 81-93 | 1.58 | 0.56 | 0.717 | 0.675 | 3 |
| MS FCA 453 | 77-89 | 0.77 | 0.75 | 0.925 | 0.69 | 4 |

Table S2: Size of the study area and the population estimate of snow leopards and their wild ungulate prey across seven study sites.

| **Study Site** | **Study are (sq km)** | **Snow leopard population estimate (95% CI)** | **Wild ungulate population estimate (95% CI)** | **Livestock population** | **Elevation (m)** | **Grazed livestock species** | **Free-ranging livestock species** | **Wild prey species** |
| --- | --- | --- | --- | --- | --- | --- | --- | --- |
| Lingti | 240 | 8 (7-10) | 593 (547-639) | 458 | 3500 - 6000 | Cow, donkey, sheep, goat, cow-yak hybrid | Yak, horse | Blue sheep |
| Kibber | 411 | 8 (7-10) | 735 (691-779) | 3894 | 3500 - 6000 | Cow, donkey, sheep, goat, cow-yak hybrid | Yak, horse | Blue sheep, ibex |
| Tabo | 341 | 4 (4-4) | 509 (456-562) | 2795 | 3300 - 6000 | Cow, donkey, sheep, goat, cow-yak hybrid | Yak, horse | Blue sheep |
| Pin | 270 | 2 (2-2) | 184 (144-224) | 1800 | 3300 - 6000 | Cow, donkey, sheep, goat, cow-yak hybrid | Yak, horse | Ibex |
| Lossar | 219 | 1 (1-1) | 30 (16-44) | 910 | 3800 - 6000 | Cow, donkey, sheep, goat, cow-yak hybrid | Yak, horse | Ibex |
| Rumptse | 300 | 5 (4-8) | 404 (290-518) | 5864 | 3800 - 6000 | Cow, donkey, sheep, goat, cow-yak hybrid | Yak, horse | Blue sheep |
| Tost | 250 | 5 (5-5) | 236 (147-325) | 2700 | 1600 - 2400 | Sheep, goat | Horse, camel | Ibex, Argali |

Table S3: Abundance estimates of wild ungulate populations obtained using spaced double-observer surveys in seven sites. The ungulate species in each site indicated in parentheses. C is the number of groups seen in both surveys, S_1_ is the number of groups seen in first survey only, S_2_ is the number of groups seen in second survey only, Ĝ is the estimated number of groups, and *N* is the estimated population. P1 and p2 are the detection probabilities for observer one and two respectively.

| **Variable** | **Rumtse**  **(*Pseudois nayaur* & *Ovis vignei*)** | **Tost**  **(*Capra sibirica* & *O. ammon*)** | **Kibber**  **(*P. nayaur* & *C. sibirica*)** | **Tabo**  **(*P. nayaur*)** | **Pin**  **(*C. sibirica*)** | **Lossar**  **(*C. sibirica*)** | **Lingti**  **(*P. nayaur*)** |
| --- | --- | --- | --- | --- | --- | --- | --- |
| C | 13 | 15 | 37 | 25 | 7 | 1 | 25 |
| S_1_ | 3 | 9 | 9 | 14 | 3 | 1 | 6 |
| S_2_ | 3 | 6 | 8 | 6 | 4 | 1 | 7 |
| Ĝ | 19.6 | 33.38 | 55.8 | 48.2 | 15.5 | 3.5 | 39.6 |
| Var (Ĝ) | 0.88 | 6.82 | 2.76 | 5.89 | 2.75 | 0.7 | 2.4 |
| *N* | 404 | 236 | 735 | 509 | 184 | 30 | 593 |
| Var(*N*) | 3348.9 | 2297.1 | 494.0 | 706 | 400.1 | 52.9 | 546 |
| ±95% *CL* | 114.8 | 95.15 | 44.1 | 52.7 | 39.7 | 14.4 | 46.3 |
| Total area (Km²) | 300 | 250 | 411 | 341 | 497 | 219 | 240 |
| Density | 1.3 | 0.9 | 1.7 | 1.4 | 0.3 | 0.1 | 2.5 |
| P_1_ | 0.8 | 0.7 | 0.8 | 0.8 | 0.6 | 0.5 | 0.7 |
| P_2_ | 0.8 | 0.6 | 0.8 | 0.6 | 0.7 | 0.5 | 0.8 |

**Figure s1: Predicted number of livestock killed by snow leopards (per year) along gradients of livestock and wild ungulates based on the simulation model for various values of (c) prey preference and (m) strength of switching. Scenario (e & b) are based on the field estimates. Scenario (c) shows the apparent facilitation of livestock by wild prey while scenario (d) shows no impact of wild prey on livestock predation.**


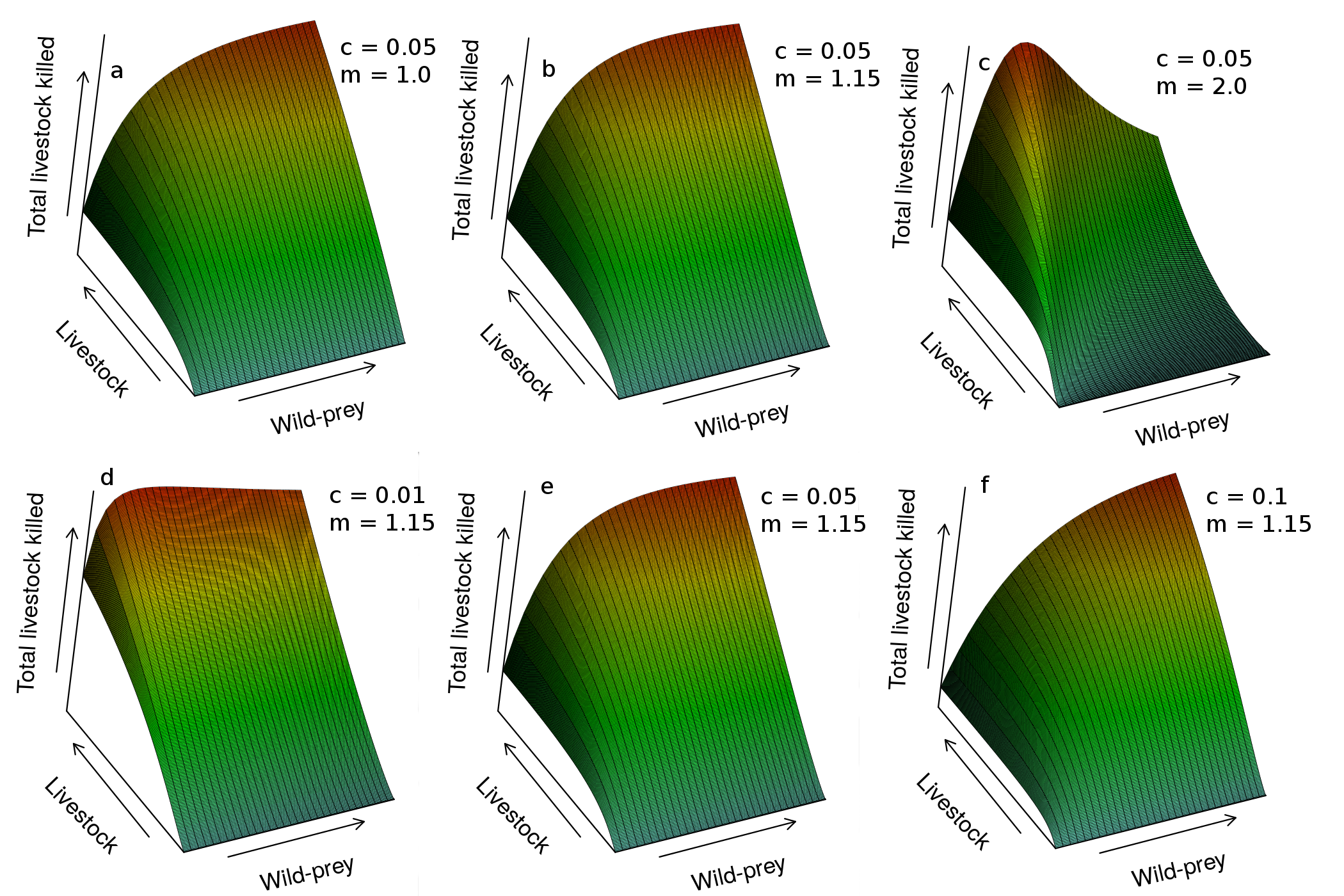


**Map:** The five sites within Himachal pradesh have been mapped in figure (a); Rumptse and Tost have been mapped in figure (b) and (c) respectively. Continuous bold lines indicate study site border. Dotted bold lines indicate the transects for scat collection. The darker contours in map (a) and (b) indicate non-habitats above 5200 meters.


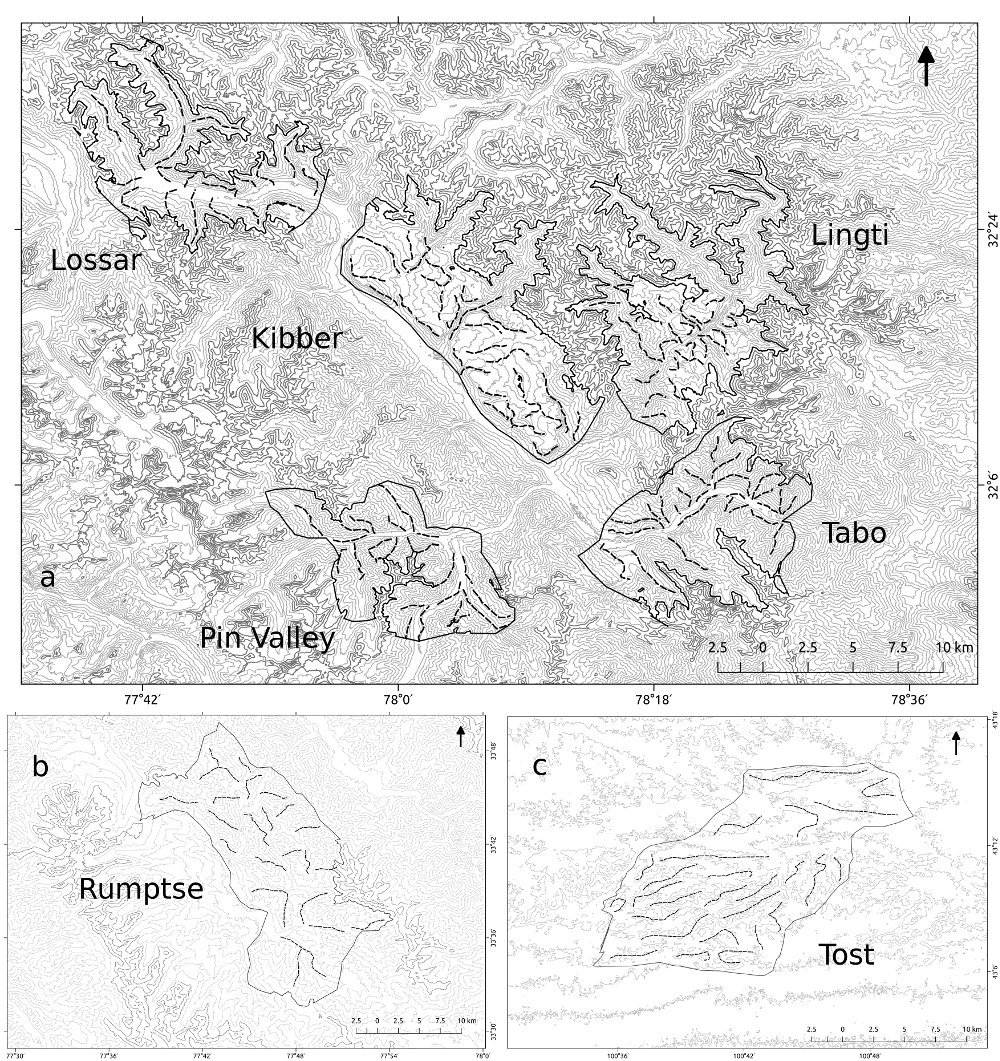


**References**

Janecka, J. E., et al. (2008) Population monitoring of snow leopards using noninvasive collection of scat samples: a pilot study. Animal Conservation, 11, 401-411.

Mondol, S., Thatte, P., Yadav, P., Ramakrishnan, U. (2012). A set of miniSTRs for population genetic analyses of tigers (*Panthera tigris*) with cross-species amplification for seven other Felidae. Conservation Genetics Resources, 4, 63-66.

Taberlet, P., (1996). Reliable genotyping of samples with very low DNA quantities using PCR. Nucleic Acids Reserch, 24, 3189-3194.

Miquel, C., Bellemain, E., Poillot, C., Bessiere, J., Durand, A., & Taberlet, P. (2006). Quality indexes to assess the reliability of genotypes in studies using noninvasive sampling and multiple-tube approach. Molecular Ecology, 6, 985–988.

Mondol, S., Karanth, K.U., Kumar, N.S., Gopalaswamy, A.M., Andheria, A., & Ramakrishnan, U. (2009). EvaluatiSon of non-invasive genetic sampling methods for estimating tiger population size. Biological Conservation, 142, 2350-2360.
